# Supplementary material for: Sexual excitation induces courtship ultrasonic vocalizations and cataplexy-like behavior in orexin neuron-ablated male mice
Source: Commun Biol. 2021 Feb 5;4:165. doi: 10.1038/s42003-021-01696-z (PMC7864915; doi:10.1038/s42003-021-01696-z)
Supplement: Supplementary file 2 — Description of Additional Supplementary Files [file 42003_2021_1696_MOESM2_ESM.pdf]

Description of Additional Supplementary Files

File name: Supplemental Movie 1 & 2. Representative video showing sonogram of ultrasonic vocalizations (USVs) and cataplexy-like attack

Description: Upper: Scrolling sonogram. The vertical axis shows the frequency of the recorded sound and the horizontal axis shows the elapsed time. Lower: Animal behavior taken from a video camera attached to the ceiling of the soundproof box. The mouse with brown fur is an orexin neuron-ablated male mouse and the mouse with black fur is a female C57BL/6J

File name: Supplementary Data 1

Description: Dataset used to make figures reported in the paper
