# Supplementary material for: Sexual excitation induces courtship ultrasonic vocalizations and cataplexy-like behavior in orexin neuron-ablated male mice
Source: Commun Biol. 2021 Feb 5;4:165. doi: 10.1038/s42003-021-01696-z (PMC7864915; doi:10.1038/s42003-021-01696-z)
Supplement: Supplementary file 6 — Reporting Summary [file 42003_2021_1696_MOESM6_ESM.pdf]

nature research

Corresponding author(s): Tomoyuki Kuwaki

Last updated by author(s): Dec 23, 2020

Reporting Summary

Nature Research wishes to improve the reproducibility of the work that we publish. This form provides structure for consistency and transparency in reporting. For further information on Nature Research policies, see our Editorial Policies and the Editorial Policy Checklist.

Statistics

For all statistical analyses, confirm that the following items are present in the figure legend, table legend, main text, or Methods section.

n/a	Confirmed	
☐	☒	The exact sample size (*n*) for each experimental group/condition, given as a discrete number and unit of measurement
☐	☒	A statement on whether measurements were taken from distinct samples or whether the same sample was measured repeatedly
☐	☒	The statistical test(s) used AND whether they are one- or two-sided<br>*Only common tests should be described solely by name; describe more complex techniques in the Methods section.*
☐	☒	A description of all covariates tested
☐	☒	A description of any assumptions or corrections, such as tests of normality and adjustment for multiple comparisons
☐	☒	A full description of the statistical parameters including central tendency (e.g. means) or other basic estimates (e.g. regression coefficient) AND variation (e.g. standard deviation) or associated estimates of uncertainty (e.g. confidence intervals)
☐	☒	For null hypothesis testing, the test statistic (e.g. *F*, *t*, *r*) with confidence intervals, effect sizes, degrees of freedom and *P* value noted<br>*Give P values as exact values whenever suitable.*
☒	☐	For Bayesian analysis, information on the choice of priors and Markov chain Monte Carlo settings
☒	☐	For hierarchical and complex designs, identification of the appropriate level for tests and full reporting of outcomes
☒	☐	Estimates of effect sizes (e.g. Cohen's *d*, Pearson's *r*), indicating how they were calculated

Our web collection on statistics for biologists contains articles on many of the points above.

Software and code

Policy information about availability of computer code

Data collection	SpectoLibellus2D (Katou 261 Acoustics Consultant Office, Kanagawa, Japan) was used in Analog-to-digital conversion of ultrasound vocalizations.<br>Video capture function in LabChart (ADInstruments Inc., Bella Vista, NSW, Australia) was used to video-recording.
Data analysis	Prism software v.6 (GraphPad) was used

For manuscripts utilizing custom algorithms or software that are central to the research but not yet described in published literature, software must be made available to editors and reviewers. We strongly encourage code deposition in a community repository (e.g. GitHub). See the Nature Research guidelines for submitting code & software for further information.

Data

Policy information about availability of data

All manuscripts must include a data availability statement. This statement should provide the following information, where applicable:
- Accession codes, unique identifiers, or web links for publicly available datasets
- A list of figures that have associated raw data
- A description of any restrictions on data availability

The summary statistics are available within the article. The data that support the findings of this study are available from the corresponding author upon reasonable request.

Field-specific reporting

Please select the one below that is the best fit for your research. If you are not sure, read the appropriate sections before making your selection.

- [x] Life sciences
- [ ] Behavioural & social sciences
- [ ] Ecological, evolutionary & environmental sciences

For a reference copy of the document with all sections, see nature.com/documents/nr-reporting-summary-flat.pdf

Life sciences study design

All studies must disclose on these points even when the disclosure is negative.

Sample size	No sample size calculation was performed. Sample size was chosen from our previous study examining the number of cataplexy bouts in the ORX-AB mice (Su et al., Sci Resp 10, 4958).
Data exclusions	No data were excluded from the analyses.
Replication	All attempts at replication were successful.
Randomization	In the experiment-1, the order of light/dark recording sessions was randomized (3 mice experienced the light period first and a separate 3 mice had dark period recording first) .<br>In the experiment-2, 3 ORX-AB mice were first examined with female encounters and the remaining 3 ORX-AB mice had male encounters. After an interval of over 3 days, the groups were again examined following an encounter with the opposite sex they initially encountered during the first examination.
Blinding	Cataplexy behavior and ultrasonic vocalizations were analyzed in a blinded manner to the treatments during data collection.

Reporting for specific materials, systems and methods

We require information from authors about some types of materials, experimental systems and methods used in many studies. Here, indicate whether each material, system or method listed is relevant to your study. If you are not sure if a list item applies to your research, read the appropriate section before selecting a response.

Materials & experimental systems

n/a	Involved in the study	
[ ]	[x]	Antibodies
[x]	[ ]	Eukaryotic cell lines
[x]	[ ]	Palaeontology and archaeology
[ ]	[x]	Animals and other organisms
[x]	[ ]	Human research participants
[x]	[ ]	Clinical data
[x]	[ ]	Dual use research of concern

Methods

n/a	Involved in the study	
[x]	[ ]	ChIP-seq
[x]	[ ]	Flow cytometry
[x]	[ ]	MRI-based neuroimaging

Antibodies

Antibodies used	Goat anti-MCH, Santa Cruz Biotechnology Cat#sc-14507<br>Rabbit anti-orexin, Peptide Institute Cat#14346-v<br>CF568-labelled Donkey anti-Rabbit IgG Biotium Cat#20098<br>CF488-labelled Donkey anti-Goat IgG Biotium Cat#20016
Validation	Specificity of anti orexin antibody was confirmed in this manuscript showing no staining in orexin neuron ablated mice.<br>Information about anti MCH is available at https://www.citeab.com/antibodies/813603-sc-14507-pro-mch-antibody-e-16

Animals and other organisms

Policy information about studies involving animals; ARRIVE guidelines recommended for reporting animal research

Laboratory animals	Mice: TetO-DTA: B6.Cg-Tg (tetO DTA) 1Gfi/J The Jackson Laboratory 008468 and<br>Mice: ORX-tTA: B6;129-Tg (ORX tTA) Tabuchi S., et al. J Neurosci 34, 6495-6509<br>were mated to make orexin neuron-ablated mice (ORX-AB). Male ORX-AB mice (8-12 wks old) were used in this study.<br>Mice: C57BL/6J Clea Japan (both males and females, 8-12 wks old) were also used.
Wild animals	The study did not involve wild-animals.

Field-collected samples	The study did not involve samples collected from the field.
Ethics oversight	The study protocol was approved by the Experimental Animal Research Committee of Kagoshima University (MD17105).

Note that full information on the approval of the study protocol must also be provided in the manuscript.
